# Concentrated urine, low urine flow, and postoperative elevation of plasma creatinine: A retrospective analysis of pooled data

**DOI:** 10.1371/journal.pone.0290071

**Published:** 2023-08-17

**Authors:** Robert G. Hahn, Laurence Weinberg, Yuhong Li, Hans Bahlmann, Rinaldo Bellomo, Patrick Y. Wuethrich

**Affiliations:** 1 Karolinska Institutet at Danderyds Hospital (KIDS), Stockholm, Sweden; 2 Department of Anesthesia, Austin Hospital; Melbourne, Australia; 3 Department of Critical Care, The University of Melbourne, Melbourne, Australia; 4 Department of Anesthesiology, Shulan International Hospital, Shuren University, Hangzhou, 3100004, Zhejiang Province, PR of China; 5 Department of Anesthesiology and Intensive Care in Linköping, Department of Biomedical and Clinical Sciences, Linköping University, Linköping, Sweden; 6 Department of Intensive Care, Austin Hospital, Melbourne, Australia; 7 Department of Critical Care, The University of Melbourne; Melbourne, Australia; 8 Department of Anesthesiology and Pain Medicine, Inselspital, Bern University Hospital, University of Bern, Bern, Switzerland; Universidad Mayor de San Simon Facultad de Medicina, PLURINATIONAL STATE OF BOLIVIA

## Abstract

Elevations of plasma creatinine are common after major surgery, but their pathophysiology is poorly understood. To identify possible contributing mechanisms, we pooled data from eight prospective studies performed in four different countries to study circumstances during which elevation of plasma creatinine occurs. We included 642 patients undergoing mixed major surgeries, mostly open gastrointestinal. Plasma and urinary creatinine and a composite index for renal fluid conservation (Fluid Retention Index, FRI) were measured just before surgery and on the first postoperative morning. Urine flow was measured during the surgery. The results show that patients with a postoperative increase in plasma creatinine by >25% had a high urinary creatinine concentration (11.0±5.9 *vs*. 8.3±5.6 mmol/L; *P*< 0001) and higher FRI value (3.2±1.0 *vs*. 2.9±1.1; *P*< 0.04) already before surgery was initiated. Progressive increase of plasma creatinine was associated with a gradually lower urine flow and larger blood loss during the surgery (Kruskal-Wallis test, *P*< 0.001). The patients with an elevation > 25% also showed higher creatinine and a higher FRI value on the first postoperative morning (*P*< 0.001). Elevations to > 50% of baseline were associated with slightly lower mean arterial pressure (73 ± 10 *vs*. 80 ± 12 mmHg; *P*< 0.005). We conclude that elevation of plasma creatinine in the perioperative period was associated with low urine flow and greater blood loss during surgery and with concentrated urine both before and after the surgery. Renal water conservation-related mechanisms seem to contribute to the development of increased plasma creatinine after surgery.

## Introduction

Plasma creatinine (pCr) is the most widely used index of impaired kidney function. Elevation of pCr occurs in approximately one-third of the patients which have undergone major surgery, and 10% reach either a 50% increase or an increase of 26.4 μmol l^-1^ which fulfils the criterion for Stage I of acute kidney injury (AKI) [1–3]. These events are associated with a statistically increased risk of long-term morbidity and mortality [4–6]. However, most increases are transient, being highest on the first or second postoperative day, and there is no accepted pathophysiological mechanism to explain them. Nonetheless, postoperative elevations in pCr are still considered a complication because they are believed to reflect reversible surgery-induced functional or morphological injury to the kidneys. Other biomarkers have been proposed, such as neutrophil gelatinase-associated lipocalin (NGAL) and chemokine (C-C motif) ligand 14 (CCL14), but none of them has yet replaced pCr as key indicator of AKI [7–9]. Therefore, a rise in pCr still serves as the fundamental indicator of postoperative AKI and might even be the only factor that determines whether a clinical trial recommends a treatment or not [10].

We have previously shown that concentrated urine is overrepresented in patients undergoing surgery and that a minor rise in pCr frequently occurs during the night before surgery [11]. Therefore, postoperative elevations of pCr may reflect pre-existing limitations of glomerular filtration that are exacerbated by the fluid retention that occurs during surgery.

We aimed to further explore the relationships between concentrated urine and postoperative pCr by summarizing data from 642 patients from eight studies performed in four countries (Australia, China, Sweden, and Switzerland), where data were collected using a similar methodology. This study aimed to test the hypothesis that a robust statistical relationship exists between urinary concentration, low urine flow, and pCr increase after surgery.

## Materials and methods

We pooled data from eight prospective studies conducted between 2011 and 2019 where pCr changes were measured immediately before surgery and in the first postoperative morning [12–19]. The urinary concentration of creatinine (uCr) was measured at the same time (90%), and a robust index of renal water conservation was obtained by calculating the Fluid Retention Index (FRI). These studies primarily included open or laparoscopic abdominal procedures, with one study including hip fracture operations [13]. The exclusion criteria were age <18 years and severe cardiac lung, hepatic, or kidney disease (CKD < 3b). The investigators agreed to participate in the secondary aggregated analyses. The reporting adhered to the STROBE checklist.

### Ethics

All studies were carried out according to the Declaration of Helsinki and approved by the appropriate Ethics Committee. The patients were recruited and studied between July 7, 2012, and November 28, 2019. They signed an approval for participation after receiving verbal and written information about the purpose and content of each study. We included all patients in the 8 studies for whom both a pre- and a postoperative pCr analysis was available. The manuscript was authored based in de-identified data during 2021 and 2022.

### Measurements

The patients arrived at the operating theatre between 7 am and 9 am after fasting overnight. General anesthesia with tracheal intubation was used as conventional anesthesia. Urine was measured via an indwelling catheter, and the urine volume was measured from the onset of anesthesia until the end of the surgery; in some instances, urine collection continued until discharge from the postoperative care unit.

Fluid therapy consisting of lactated Ringer’s solution was supplemented with 6% hydroxyethyl starch (130/0.4), albumin (20%), or blood products at the clinicians’ discretion.

Monitoring included pulse oximetry, heart rate measurement, invasive arterial pressure measurement, and electrocardiography. Data were recorded every 5–15 min and the mean individual value of the mean arterial pressures (MAP) was calculated and reported. Blood loss was estimated based on the amount of blood in the suction tubes and weighed sponges at the end of the study. All excreted urine during the surgery was measured.

Preoperative blood and urine sampling was performed the day before surgery or just before the induction of anesthesia. Blood and urine samples were also collected on the morning after surgery. The pCr and concentration was measured in both blood samples. The urine samples were analyzed for uCr (enzymatic method), osmolality (freezing point depression), and specific gravity (refractometry) by the certified clinical chemistry laboratory at each hospital. Urine color was assessed by visual estimation using a published color chart [20].

These urinary biomarkers represent metabolic waste substances that occur in higher urine concentrations when the kidneys conserve water. The rationale is that end products from the metabolism of mostly erythrocytes and muscle are excreted at a relatively constant rate throughout the day regardless of the turnover of water.

### Fluid retention index (FRI)

The FRI is a composite measure of renal water retention based on the above four urinary biomarkers, according to the scheme shown in **Table 1** [21]. These biomarkers are inter-correlated in an exponential fashion [22]. The urinary creatinine concentration shows a greater response to variations in habitual water ingestion than the other biomarkers, in particular when the intake is low [23]. However, all four biomarkers can still be calibrated into each other to generate a robust index that is less sensitive to outliers, as individual biomarkers may occasionally be distorted due to diet and medication. The scores assigned to each biomarker are summarized, and the mean taken as the FRI value. Dehydration by ≥ 5% of the body weight corresponds to FRI ≥ 4 [21,24,25]. uCr was included in the FRI score but was also reported separately, as pCr is the reference outcome measure.

**Table 1:** Description of the four dimensions of the Fluid Retention Index (FRI).

Score	1	2	3	4	5	6
Specific gravity	≤1.005	1.010	1.015	1.020	1.025	1.030
Osmolality (mOsmol kg^-1^)	<250	250–450	450–600	600–800	800–1000	>1000
Creatinine (uCr; mmol L^-1^)	<4	4–7	7–12	12–17	17–25	>25
Color (shade)	1	2	3	4	5	6

The FRI has been evaluated in 57 healthy volunteers aged 17–69 of both genders who performed recreational exercise during a median time of 90 min; the FRI value changed by 31% in response to a loss of body water amounting to 1% of the body weight (*P*< 0.001). The correlation coefficients for the relationships between the four variables varied between 0.71 and 0.84 [21]. The correlation coefficients were essentially the same when relationships were re-evaluated in 300 volunteers not subjected to exercise [22].

### Statistics

Data showing a normal distribution are presented as the mean ± standard deviation (SD). Differences between groups were evaluated by one-way analysis of variance (ANOVA) followed by the Scheffé *post hoc* test, as appropriate, when more than two groups were compared.

Correlations between perioperative variables shown to differ depending on the pCr change were studied by multivariate logistic regression analysis using the routine implemented in SPSS version 28.0.0 for Mac (IBM Corp., Armonk, NY).

Data showing a skewed distribution are reported as the median (25th–75th percentile limits), and differences were assessed using the Mann-Whitney U test (two groups) or the Kruskal-Wallis test followed by the pairwise *post hoc* test implemented in SPSS. Changes over time were assessed using the Wilcoxon matched-pair test (two measurements). *P*< 0.05 was considered statistically significant.

## Results

The analysis included 642 patients aged 62 ± 13 years (mean ± SD) with a body weight of 72±16 kg. The operating time was 4.2 ± 2.1 h. Basic demographic information on the different studies is summarized in **Table 2.**

**Table 2:** Key data from the analyzed studies.

Reference	12	13	14	15	16	17	18	19
Type of surgery	Gastrointestinal, 34% open	Hip fracture, spinal	Open abdominal	Mixed surgeries	Esophageal, open	Open urologic, cancerous	Gastrointestinal,83% open	Colorectal cancer, laparoscopic
Country	China	Sweden	Sweden	Sweden	Sweden	Switzerland	Australia	China
Year	2014	2015	2016	2017	2019	2021	2021	2022
N	84	38	79	9	56	185	65	126
Females (%)	6	47	55	100	22	22	49	32
Age (years)	58 ± 12	78 ± 11	63 ± 13	50 ± 8	65 ± 9	62 ± 14	62 ± 11	61 ± 11
Body weight (kg)	59 ± 9	68 ± 12	76 ± 15	72 ± 20	66 ± 9	82 ± 16	77 ± 16	63 ± 9
Hypertension (%)	-	35	28	22	37	48	-	35
Diabetes (%)	-	14	15	9	0	11	21	4
ASA class (1, 2, 3)	-	0, 38, 62	28, 60, 12	56, 44, 0	19, 56, 24	49, 49, 2	44, 54, 2	12, 86, 2
Operating time (h)	3.3 ±1.2	1.1 ± 0.6	3.4 ± 2.1	1.4 ± 0.7	7.0 ± 1.4	4.2 ± 1.6	4.2 ± 1.6	5.3 ± 2.4
Blood loss (mL)	50 (50–50)	300 (200–450)	275 (100–575)	275 (200–400)	400 (250–700)	400 (250–703)	100 (50–500)	100 (50–100)
pCr before surgery (μmol L^-1^)	70 ± 15	85 ± 44	72 ± 17	65 ± 8	74 ± 17	90 ± 29	69 ± 15	69 ± 13
pCr after surgery (μmol L^-1^)	61 ± 14	85 ± 43	83 ± 62	58 ± 7	69 ± 22	106 ± 47	76 ± 30	70 ± 14
pCr change (% in Groups 1–4)	82, 18, 0, 0	61, 23, 13, 3	58, 28, 5, 9	89, 11, 0, 0	66, 27, 5, 2	25, 47, 16, 12	48, 26, 14, 12	48, 48, 4, 0

Data are the mean ± SD except blood loss, which is median (25^th^-75^th^ percentile range).

The change in pCr from just before surgery to the first postoperative day was divided into four ranges: (**1**) decrease, (**2**) increase up to 25%, (**3**) increase by 26–50%, and (**4**) increase by >50%. The last group, which fulfilled the criteria for AKI Stage I, constituted 6.1% of the cohort. This fraction increased to 8.6% when the alternative criterion i.e., a perioperative increase in pCr by ≥ 26.5 μmol l^-1^, was applied.

### Preoperative data

The four groups with different plasma creatinine responses to surgery did not differ in age or baseline plasma creatinine; body weight was higher in Groups 3 and 4 than in Groups 1 and 2 (pooled weights 83 ± 15 kg *vs*. 70 ± 15 kg, *P*< 0.001; **Table 3**).

**Table 3:** Results of the pooled analysis of physiological and biochemical data.

Perioperative change in plasma creatinine (to POD 1), ratio						
	(1) Decrease	(2) 0 to +25%	(3) +26 to +50%	(4) > 50%	P-value	*Post hoc* test *P*< 0.05 is shown
Number	320	227	56	39		
Age (years)	62 ± 12	64 ± 13	63 ± 15	63 ± 16	0.58	
Body weight (kg)	68 ± 15	72 ± 15	80 ± 14	86 ± 16	0.001	3, 4 > 1, 2
Operating time (h)	4.1 ± 2.1	4.4 ± 2.0	4.6 ± 2.2	4.5 ± 2.1	0.20	
uCr (mmol l^-1^)						
Before surgery	7.9 ± 5.2	8.9 ± 5.9	11.4 ± 5.9	10.2 ± 5.7	0.001	3 > 1
After surgery	7.9 ± 5.8	9.6 ± 7.0	12.4 ± 7.1	14.0 ± 7.1	0.001	3, 4 > 1, 2
uOsmolality (mosmol kg^-1^)						
Before surgery	483 ± 221	516 ± 220	553 ± 158	502 ± 197	0.22^1^	
After surgery	557 ± 235	635 ± 239	658 ± 205	628 ± 213	0.007	2, 3 > 1
uSpecific gravity						
Before surgery	1.016 ± 0.007	1.016 ± 0.007	1.017 ± 0.006	1.015 ± 0.006	0.53	
After surgery	1.020 ± 0.009	1.021 ± 0.010	1.022 ± 0.010	1.023 ± 0.008	0.48	
FRI score						
Before surgery	2.9 ± 1.1	3.0 ± 1.1	3.4 ± 0.9	3.1 ± 1.1	0.001	3, 4 > 1
After surgery	3.3 ± 1.1	3.7 ± 1.0	4.1 ± 1.0	4.0 ± 1.1	0.001	2–4 > 1
Urine volume (ml)	405 (200–648)	300 (100–548)	163 (100–275)	135 (50–400)	0.001	1 > 2–4
Urine flow (ml min^-1^)	1.5 (0.9–2.5)	1.1 (0.5–2.0)	0.7 (0.4–1.3)	0.6 (0.4–1.3)	0.001	1 > 2–4, 2 > 4
MAP (mmHg)	80 ± 13	80 ±10	80 ± 9	73 ± 10	0.04	2 > 4
Blood loss (ml)	100 (50–400)	200 (60–500)	275 (100–500)	350 (110–1150)	0.001	2–4 > 1; 4 > 2
pCr (μmol l^-1^)						
Before surgery	77 ± 25	76 ± 21	78 ± 28	82 ± 28	0.56	
After surgery	66 ± 23	84 ± 23	105 ± 38	168 ± 91	0.001	All groups differ

Data are the mean ± SD or median (25th-75th percentile limits). FRI = Fluid Retention Index. NS = not significant. P-values was obtained with ANOVA or the Kruskal-Wallis test. *Post hoc* analysis by the Scheffé *post hoc* test is reported when one-way ANOVA had been applied. Urine volume, urine flow, preoperative FRI, and blood loss were evaluated by the Kruskal-Wallis test followed by the *post hoc* test implemented in SPSS.

^1^ *P*< 0.05 with Kruskal-Wallis test.

The urinary creatinine concentration before surgery was higher (11.0 ± 5.9 *vs*. 8.3 ± 5.6 mmol l^-1^; *P*< 0.001) as was the FRI value (3.2 ± 1.0 *vs*. 2.9 ± 1.1; *P*< 0.04) in the patients who later developed an increase in pCr.

### Data during surgery

**Fig 1:**
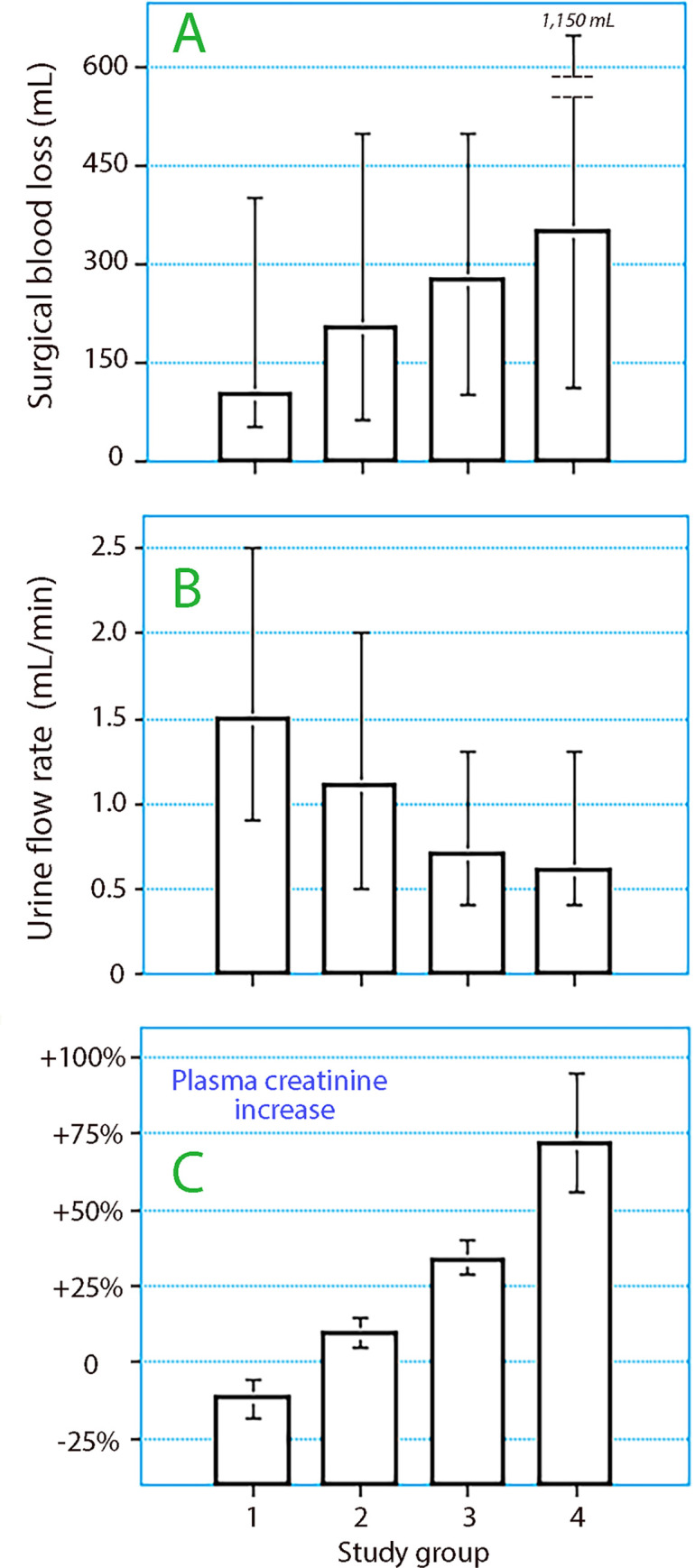
**(A)** The blood loss during surgery and **(B)** the urine flow rate during the surgery depending on **(C)** the degree of perioperative change in pCr. Data are the median and 25th-75th percentiles.

As shown in **Table 3**, despite similar operative times, the urinary output and urine flow decreased with a stepwise increase in pCr (Kruskal-Wallis test, both *P*<0.001). For example, the median urine flow rate during surgery was 1.5 (0.9–2.5) ml min^-l^ in Group 1, 1.1 (0.5–2.0) ml min^-l^ in Group 2, 0.7 (0.4–1.3) ml min^-l^ in Group 3, and 0.6 (0.4–1.3) ml min^-1^ in Group 4. Data were markedly skewed as illustrated by the wide 25^th^-75^th^ percentile range. By contrast, blood loss increased with stepwise changes in plasma creatinine levels (*P*< 0.001, **Fig 1**). Patients in Group 4 had a lower MAP than the others (73 ± 10 *vs*. 80 ± 12 mmHg; *P*< 0.005).

Logistic regression was performed to study whether the four factors for which ANOVA showed a between-group difference independently predicted the change in pCr. However, singularities in the Hessian matrix in multinominal regression suggested that Groups 1–2 and 3–4 should be merged. The analysis then showed that uCr before surgery (*P*< 0.03), the urine flow (*P*< 0.02), and the logarithm-transformed blood loss (*P*< 0.02) but not MAP served as independent predictors of an increase in pCr by > 25%.

### Postoperative data

**Fig 2:**
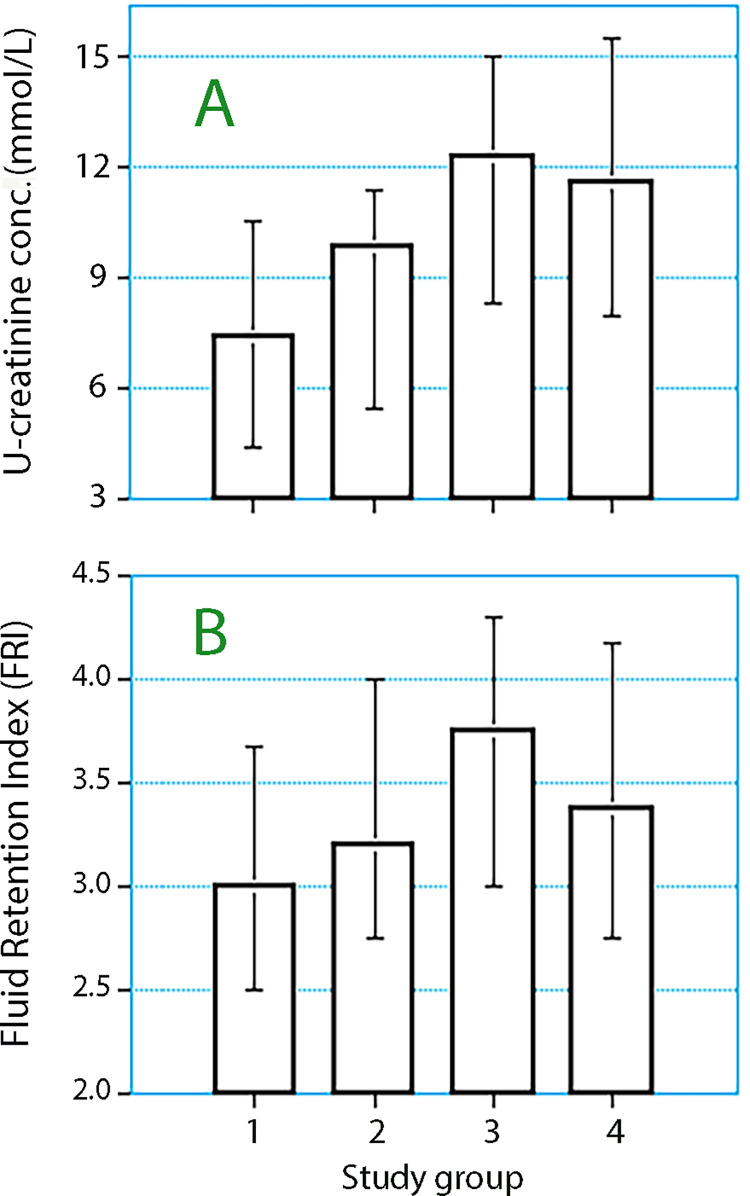
The mean value of the pre- and postoperative measurements of **(A)** The urinary creatinine (uCr) concentration and **(B)** the Fluid Retention Index (FRI) depending on the perioperative change in pCr (Study groups 1 to 4). Data are the median and 25th-75th percentiles.

Overall, uCr and FRI showed the same trends in the first postoperative morning as they did immediately before surgery. These indices of concentrated urine showed higher values with increased pCr levels during the perioperative period (P< 0.001; Table 3, Fig 2).

The postoperative uCr was higher than the preoperative concentration in Groups 3 and 4 (from 11.0 ± 5.9 to 13.0 ± 7.0 mmol l^-1^; *P* = 0.041) as was also the FRI value (from 3.5 ± 1.1 to 4.0 ± 1.0; *P*< 0.002). The perioperative change in uCr did not reach statistical significance in Groups 1 and 2 (*P* = 0.08), but the FRI increased significantly (from 2.9 ± 1.1 to 3.5 ± 1.1; *P*< 0.0001).

## Discussion

### Key findings

A postoperative increase in pCr is considered a surgical complication based on the belief that it represents acute functional or organic damage to the kidneys. The present study challenges this view by identifying circumstances under which a postoperative increase in pCr is more likely to be a natural consequence of excessive fluid retention and not necessarily indicating kidney damage. Our analysis shows that postoperative elevations in pCr were more likely if patients had concentrated urine before surgery, as evidenced by higher uCr and FRI. Elevations of pCr were further associated with lower urine flow, greater blood loss during surgery, and more concentrated urine in the first postoperative morning. MAP was also significantly lower in patients with the greatest elevation in pCr levels, but the magnitude of the difference was small.

### Postulated mechanisms

High uCr and high FRI before the surgery is consistent with "concentrated urine", which is common in the general population [22] and is associated with low habitual intake of water [23,26,27], elevated plasma vasopressin [28], and slightly lower glomerular filtration rate (GFR) [29]. A retrospective review of data from our laboratory supports that uCr >7 mmol l^-1^ had an ioxhexol-measured GFR being 20% lower than other patients with lower uCr [30].

Fluid retention becomes aggravated during surgery due to the anesthesia-induced reduction of MAP, which unloads the baroreceptors and thereby activates the sympathetic nerves leading to the kidneys. Such activation increases sodium and water reabsorption, induces renin secretion, and further reduces the GFR [31,32]. This chain of events is supported by experimental anesthesia (without surgery) during which plasma renin but not the vasopressin concentration increases [33,34] while diuretic response to crystalloid fluid is strongly impaired (-85%) [35,36]. The urine becomes more concentrated when the urine flow falls below 1 ml min^-1^ during surgery [37], which occurred in 38% of the patients in this study (**Table 3**). However, the fluid retention due to low MAP during surgery resolves soon after awakening from general anesthesia [38].

In the present study, renal water conservation was still strong on the first postoperative morning. Concentrated urine due to low habitual intake of water reacts slowly to increased ingestion of fluid (>1 week) [23] except for dramatic changes [39,40]. Here, the postoperative fluid therapy was not large enough to reverse the kidney´s efforts to conserve water. However, water-sparing hormones (aldosterone, cortisol etc.) excreted due to “surgical stress” may have contributed to the postoperative fluid retention.

Pre-existing limitations of kidney function are likely to make patients more susceptible to developing AKI as defined based on an elevation of pCr. In one of our studies, most patients who developed Stage I AKI after surgery had a small rise in pCr during the immediate *preoperative* period [11]. Regardless of habitual water intake, patients being close to their maximum capacity to excrete creatinine are probably more likely than others to develop AKI in response to transient anesthesia-induced fluid retention. It might be for this reason, rather than because of surgery, that these patients have higher long-term morbidity in cardiovascular disease [4–6].

### Kidney damage?

The uCr concentration is the sum of the glomerular filtration, renal water conservation, and tubular secretion of creatinine. Creatinine is filtered freely in the glomeruli; however, tubular reabsorption of water concentrates creatinine by a factor of approximately 100. The maximal threshold for excretion is probably only two to three times higher, although the limit is certainly lower when there is pre-existing kidney injury. Tubular secretion may increase creatinine excretion by 10–30%.

The uCr concentration and other FRI components are strongly intercorrelated in a mildly non-linear fashion [22,23] and any departure from their expected relationship could indicate temporary reduction of the GFR. We have reported such discrepancies during surgery in two of our studies in which urine was also assessed during that period [17,19]. Here, uCr during surgery was lower than expected in patients who had concentrated urine before surgery [16] and in those who developed a postoperative increase in pCr [18].

Our postoperative data support that the kidneys strove to filter the creatinine that had not been adequately excreted during the surgery, even in patients who developed Stage I AKI. Elevation of pCr due to functional or organic damage to kidney cells is expected to co-exist with impaired ability to concentrate creatinine, but the ability to concentrate creatinine after surgery was well preserved in this study.

### Blood flow and AKI

Reduced blood flow to the kidneys is a key factor in the development of AKI [41]. In sheep the blood flow to the kidneys decreased by 2/3 from general anesthesia alone [42] and is likely to be further aggravated by pre-existing dehydration and hypovolemia. Acute dehydration of 5–6% of the body weight, which is indicated at the far right of the FRI scale, is associated with a risk of anuria in conscious humans. In the dog dehydration by 9% of the body weight followed by a diuretic and peritoneal dialysis that increased the hematocrit by 60% which caused anuria and periods of intermittent blood flow to the inner cortex and possibly caused anoxic damage [43]. Induction of central hypovolemia in the pig reduced the blood flow to the kidneys by 90% while the reduction of the blood flow to the splanchnic region was only half as great [44]. Restrictive fluid therapy during surgery increases the likelihood of a postoperative rise in plasma creatinine [2,10] but if the elevation is due to impaired blood flow or excessive fluid retention is unclear.

### Limitations and strengths

The diagnostic tools used to identify acute kidney injury are questioned. The current criteria based on pCr elevation and urine output are not optimal [45]. Whilst it appears to be promising that novel and more specific biomarkers may be able to predict AKI [7–9] it is however unclear whether some of the commercially available biomarker kits adequately indicate physiological or morphological kidney injury [46,47].

The FRI value is a composite index based on four biomarkers of concentrated urine that also includes uCr, which was studied separately. The other two objective indices, urine osmolality and urine-specific gravity, showed the same overall trend as uCr but the between-group differences were smaller and usually not statistically significant (Table 3). This can be understood from the greater differences in uCr compared to the other two biomarkers for variations in habitual intake of water [23] and the non-linearity between in that has been observed in a cross-sectional analysis [22]. Some of the non-linearity between the biomarkers have been rectified in the construction of the FRI scale (Table 1).

Previous studies have shown a relationship between MAP and postoperative AKI [48–50]. Our data partially support this relationship, but differences in MAP between the groups and the recorded periods of very low pressure were small. Therefore, preoperative renal water conservation probably caused the main difference between the study groups. The surgical blood loss also differed between the groups, but the bleeding in the group with plasma creatinine elevation in the highest range was still not severe (median, 350 ml). However, the variables indicating renal water conservation hardly differed between Groups 3 and 4, which leaves MAP, blood loss, and possibly body weight to be the remaining factors that promoted a rise in pCr of >50% rather than of 25–50%.

All studies did not contribute with data on all variables. The study of esophageal surgery did not measure FRI or uCr [16] and another study provided no postoperative data on these variables [14]. The number of patients contributing with uCr and urine-specific gravity was 506 while data on urine osmolality was available in 496 patients.

No follow-up was conducted to determine whether the increase in pCr persisted in any of the studies. We can assume that this is not common, but permanent kidney injury might have occurred in a few patients.

Strengths of this report include that the eight included studies were prospective and that patients with severe cardiac lung, hepatic, or kidney disease were excluded.

## Conclusions

Postoperative elevation of pCr correlated with low urine flow during surgery and with concentrated urine, both before and after surgery. A likely triad of causes include low dietary intake of water or preoperative dehydration, increased adrenergic activity in the kidneys during surgery, and a low pre-existing functional threshold for excretion of creatinine.

## Supporting information

S1 Checklist (DOCX)

S2 Checklist (DOC)

S1 File The original data. (XLS)

## References

[1] Bernardi MH, Ristl R, Neugebauer T, Hiesmayr MJ, Druml W, Lassnigg A. Very early changes in serum creatinine are associated with 30-day mortality after cardiac surgery. *Eur J Anaesthesiol* 2020; 37:898–907. 32371831 10.1097/EJA.0000000000001214

[2] Furrer MA, Schneider MP, Löffel LM, Burkhard FC, Wuethrich PY. Impact of intra-operative fluid and noradrenaline administration on early postoperative renal function after cystectomy and urinary diversion: A retrospective observational cohort study. *Eur J Anaesthesiol* 2018; 35:41–49. doi: 10.1097/EJA.0000000000000808 29652680

[3] Gomelsky A, Abreo K, Khater N, et al. Perioperative acute kidney injury: Stratification and risk reduction strategies. *Best Pract Res Clin Anaesthesiol* 2020; 34:167–182. doi: 10.1016/j.bpa.2020.04.003 32711827

[4] Boyer N, Eldridge J, Prowle JR, Forni LG. Postoperative AKI. *Clin J Am Soc Nephrol* 2022; 17:1535–1545. 35710717 10.2215/CJN.16541221 PMC9528271

[5] Romagnoli S, Ricci Z, Ronco C. Perioperative acute kidney injury: prevention, early recognition, and supportive measures. *Nephron* 2018; 140:105–110. doi: 10.1159/000490500 29945154

[6] Zarbock A, Koyner JL, Hoste EAJ, Kellum JA. Update on perioperative acute kidney injury. Anesth Analg 2018; 127:1236–1245. doi: 10.1213/ANE.0000000000003741 30138176

[7] Mårtensson J, Martling CR, Bell M. Novel biomarkers of acute kidney injury and failure: clinical applicability. *Br J Anaesth* 2012; 109:843–50. doi: 10.1093/bja/aes357 23048068

[8] Ilaria G, Kianoush K, Ruxandra B, et al. Clinical adoption of Nephrocheck® in the early detection of acute kidney injury. *Ann Clin Biochem* 2020; 58:6–15. 33081495 10.1177/0004563220970032

[9] Hoste E, Bihorac A, Al-Khafaji A, et al. RUBY Investigators. Identification and validation of biomarkers of persistent acute kidney injury: the RUBY study. Intensive Care Med 2020; 46:943–953. doi: 10.1007/s00134-019-05919-0 32025755 PMC7210248

[10] Myles PS, Bellomo R, Corcoran T, et al; Australian and New Zealand College of Anaesthetists Clinical Trials Network and the Australian and New Zealand Intensive Care Society Clinical Trials Group. Restrictive versus liberal fluid therapy for major abdominal surgery. *N Engl J Med* 2018; 378:2263–2274. 29742967 10.1056/NEJMoa1801601

[11] Löffel LM, Engel DA, Beilstein CM, Hahn RG, Furrer MA, Wuethrich PY. Dehydration before major urological surgery and the perioperative pattern of plasma creatinine: A prospective cohort series. *J Clin Med* 2021; 10:5817. doi: 10.3390/jcm10245817 34945113 PMC8706637

[12] Li Y, He R, Ying X, Hahn RG. Dehydration, hemodynamics and fluid volume optimization after induction of general anesthesia. Clinics 2014; 69:809–816. doi: 10.6061/clinics/2014(12)04 25627992 PMC4286668

[13] Hahn RG. Renal injury during hip fracture surgery: an exploratory study. *Anaesthesiol Intensive Ther* 2015; 47:284–290. doi: 10.5603/AIT.a2015.0029 26037257

[14] Bahlmann H, Hahn RG, Nilsson L. Agreement between Pleth Variability Index and oesophageal Doppler to predict fluid responsiveness. *Acta Anaesthesiol Scand* 2016; 60:183–192. doi: 10.1111/aas.12632 26373826

[15] Hahn RG. Renal water conservation determines the increase in body weight after surgery; a randomized controlled trial. *Saudi J Anaesth* 2017; 11:144–51. doi: 10.4103/1658-354X.203018 28442951 PMC5389231

[16] Bahlmann H, Halldestam I, Nilsson L. Goal-directed therapy during transthoracic oesophageal resection does not improve outcome: Randomised controlled trial. Eur J Anaesthesiol 2019; 36:153–16R1. doi: 10.1097/EJA.0000000000000908 30431499

[17] Engel D, Löffel LM, Wuethrich PY, Hahn RG. Preoperative concentrated urine increases the incidence of plasma creatinine elevation after major surgery. Front Med (Lausanne) 2021; 8:699969. doi: 10.3389/fmed.2021.699969 34350198 PMC8327205

[18] Yanase F, Tosif S, Yee K, Bellomo R, Gunn K, Kim C, et al. A randomized, open-label, blinded endpoint, phase 2, feasibility, efficacy and safety trial of preoperative microvascular protection in patients undergoing major abdominal surgery. *Anesth Analg* 2021; 133:1036–1047. 34269720 10.1213/ANE.0000000000005667

[19] Li Y, He R, Hu S, Hahn RG. Renal water conservation and plasma creatinine in colorectal cancer surgery; a single-group clinical study. *Front Med* 2022; 9:837414. doi: 10.3389/fmed.2022.837414 35712088 PMC9195291

[20] Armstrong LE, Maresh CM, Castellani JW, et al. Urinary indices of hydration status. *Int J Sport Nutr* 1994; 4: 265–279. doi: 10.1123/ijsn.4.3.265 7987361

[21] Hahn RG, Waldréus N. An aggregate urine analysis tool to detect acute dehydration. *Int J Sport Nutr Exerc Metab* 2013; 23:303–311. 23994895

[22] Hahn RG, Grankvist N, Krizhanovskii C. Urinary analysis of fluid retention in the general population: a cross-sectional study. *PLoS One* 2016; 11:e0164152 doi: 10.1371/journal.pone.0164152 27764121 PMC5072703

[23] Hahn RG. Effects of diet, habitual water intake and increased hydration on body fluid volumes and urinary analysis of renal fluid retention in healthy volunteers. *Eur J Nutr* 2021; 48:310–7. doi: 10.1007/s00394-020-02275-4 32430554 PMC7900032

[24] Casa DJ, Armstrong LE, Hillman SK, et al. National athletic trainers’ association position statement: Fluid replacement for athletes. *J Athl Train* 2000; 35:212–224. 16558633 PMC1323420

[25] Popowski LA, Oppliger RA, Patrick Lambert G, Johnson RF, Kim Johnson A, Gisolf CV. Blood and urinary measures of hydration status during progressive acute dehydration. *Med Sci Sports Exerc* 2001; 33:747–753. doi: 10.1097/00005768-200105000-00011 11323543

[26] Perrier E, Vergne S, Klein A, et al. Hydration biomarkers in free-living adults with different levels of habitual fluid consumption. *Br J Nutr* 2013; 109:1678–87. doi: 10.1017/S0007114512003601 22935250 PMC3638312

[27] Johnson EC, Munoz CX, L Bellegro L, et al. Markers of the hydration process during volume modification with habitual high or low daily fluid intakes. *Eur J Appl Physiol* 2015; 115:1067–74. 25564016 10.1007/s00421-014-3088-2

[28] Johnson EC, Muñoz CX, Jimenez L, et al. Hormonal and thirst modulated maintenance of fluid balance in young women with different levels of habitual fluid consumption. Nutrients 2016; 18:8. doi: 10.3390/nu8050302 27213436 PMC4882714

[29] Kitiwan BK, Vasunilashorn SM, Baer HJ, Mukamal K, Juraschek SP. The association of urine osmolality with decreased kidney function and/or albuminuria in the United States. *BMC Nephrol* 2021; 22:306. doi: 10.1186/s12882-021-02478-9 34507548 PMC8434733

[30] Hahn RG, Nyberg Isacson M, Fagerström T, Rosvall J, Nyman CR. Isotonic saline in elderly men: an open-labelled controlled infusion study of electrolyte balance, urine flow and kidney function. Anaesthesia 2016; 71:155–162. doi: 10.1111/anae.13301 26669730

[31] Johns EJ, Kopp UC, DiBona GF. Neural control of renal function. *Compr Physiol* 2011; 1:731–767. doi: 10.1002/cphy.c100043 23737201

[32] Osborn JW, Tyshynsky R, Vulchanova L. Function of renal nerves in kidney physiology and pathophysiology. *Annu Rev Physiol* 2021; 83:429–450. doi: 10.1146/annurev-physiol-031620-091656 33566672

[33] Norberg Å, Hahn RG, Li H, Olsson J, et al. Population volume kinetics predicts retention of 0.9% saline infused in awake and isoflurane-anesthetized volunteers. *Anesthesiology* 2007; 107:24–32. doi: 10.1097/01.anes.0000268387.34758.6d 17585212

[34] Taavo M, Rundgren M, Frykholm P, et al. Role of renal sympathetic nerve activity in volatile anesthesia’s effect on renal excretory function. Function 2021; 2:zqab042. doi: 10.1093/function/zqab042 35330795 PMC8788708

[35] Hahn RG. Arterial pressure and the rate of elimination of crystalloid fluid. *Anesth Analg* 2017; 124:1824–1833. doi: 10.1213/ANE.0000000000002075 28452823

[36] Li Y, Yi S, Zhu Y, Hahn RG. Volume kinetics of Ringer´s lactate solution in acute inflammatory disease. *Br J Anaesth* 2018; 121:574–580. doi: 10.1016/j.bja.2018.04.023 30115255

[37] Hahn RG, Nemme J. Volume kinetic analysis of fluid retention after induction of general anaesthesia. *BMC Anesthesiol* 2020; 20:95. 32334513 10.1186/s12871-020-01001-1 PMC7183132

[38] Hahn RG, Olsson J. Diuretic response to Ringer´s solution is normal shortly after awakening from general anaesthesia; a retrospective kinetic analysis. *BJA Open* 2022; 2: 100013. 37588273 10.1016/j.bjao.2022.100013 PMC10430821

[39] Johnson EC, Munoz CX, L Bellegro L, et al. Markers of the hydration process during volume modification with habitual high or low daily fluid intakes. *Eur J Appl Physiol* 2015; 115:1067–1074. 25564016 10.1007/s00421-014-3088-2

[40] Johnson E, Huffman AE, Yoder H, et al. Urinary markers of hydration during 3-day water restriction and graded rehydration. *Eur J Nutr* 2019; 59:2171–2181. doi: 10.1007/s00394-019-02065-7 31428854 PMC7351875

[41] Basile DP, Anderson MD, Sutton TA. Pathophysiology of acute kidney injury. Compr Physiol 2012; 2:1303–1353. doi: 10.1002/cphy.c110041 23798302 PMC3919808

[42] Iguchi N, Kosaka J, Booth LC, Iguchi Y, Evans RG, Bellomo R, et al. Renal perfusion, oxygenation, and sympathetic nerve activity during volatile or intravenous general anaesthesia in sheep. *Br J Anaesth* 2019; 122:342–349. doi: 10.1016/j.bja.2018.11.018 30770052

[43] Kirkebo A, Tyssebothn I. Effect of dehydration of renal blood flow in dog. Ata Physiol Scand 1977; 101:257–263. 10.1111/j.1748-1716.1977.tb06006.x 596201

[44] Riddez L, Johnson L, Hahn RG. Early hemodynamic changes during uncontrolled intra-abdominal bleeding. Eur Surg Res 1999; 31:19–25. doi: 10.1159/000008617 10072607

[45] Dhawan R, Chaney MA. Renal dysfunction and cardiac surgery: How can we study an undefined entity? J Cardiothorac Vasc Anesth 2022; 36:4234–4236. doi: 10.1053/j.jvca.2022.07.027 36038443

[46] Hahn RG, Yanase F, Zdolsek JH, Tosif S, Bellomo R, Weinberg L. Serum creatinine levels and Nephrocheck values with and without correction for urine dilution—a multicenter observational study. *Front* Med (Lausanne) 2022; 9:847129 35252280 10.3389/fmed.2022.847129 PMC8894808

[47] Naorungroj T, Yanase F, Bittar I, Eastwood G, Bellomo R. The relationship between Nephrocheck® test values, outcomes, and urinary output in critically ill patients at risk of acute kidney injury. *Acta Anaesthesiol Scand* 2022; 66:1219–1227. 36056749 10.1111/aas.14133

[48] Sun LY, Wijeysundera DN, Tait GA, Beattie WS. Association of intraoperative hypotension with acute kidney injury after elective noncardiac surgery. *Anesthesiology* 2015; 23:515–523. doi: 10.1097/ALN.0000000000000765 26181335

[49] Salmasi V, Maheshwari K, Yang D, et al. Relationship between intraoperative hypotension, defined by either reduction from baseline or absolute thresholds, and acute kidney and myocardial injury after noncardiac surgery: a retrospective cohort analysis. *Anesthesiology* 2017; 126:47–65. doi: 10.1097/ALN.0000000000001432 27792044

[50] Löffel LM, Bachmann KF, Furrer MA, Wuethrich PY. Impact of intraoperative hypotension on early postoperative acute kidney injury in cystectomy patients—A retrospective cohort analysis. *J Clin Anesth* 2020; 66:109906. doi: 10.1016/j.jclinane.2020.109906 32615512

